# Tuberculosis lymphadenitis in Southwest Ethiopia: a community based cross-sectional study

**DOI:** 10.1186/1471-2458-12-504

**Published:** 2012-07-06

**Authors:** Gemeda Abebe, Amare Deribew, Ludwig Apers, Alemseged Abdissa, Fetene Deribie, Kifle Woldemichael, Jaffer Shiffa, Markos Tesfaye, Chali Jira, Mesele Bezabih, Abraham Aseffa, Alemayehu Bekele, Robert Colebunders

**Affiliations:** 1 Department of Medical Laboratory Sciences and Pathology, Jimma University, Jimma, Ethiopia; 2 Department of Epidemiology, Jimma University, Jimma, Ethiopia; 3 Department of Clinical Sciences, Institute of Tropical Medicine, Antwerp, Belgium; 4 Department of Internal Medicine, Jimma University, Jimma, Ethiopia; 5 Department of Psychiatry, Jimma University, Jimma, Ethiopia; 6 Department of Health Service Management, Jimma University, Jimma, Ethiopia; 7 Armauer Hansen Research Institute, Addis Ababa, Ethiopia; 8 Department of Epidemiology and Social Medicine, University of Antwerp, Antwerp, Belgium

**Keywords:** TB lymphadenitis, Prevalence, Jimma, Ethiopia

## Abstract

**Background:**

In Ethiopia where there is no strong surveillance system and diagnostic facilities are limited, the real burden of tuberculosis (TB) lymphadenitis is not well known. Therefore, we conducted a study to estimate the prevalence of TB lymphadenitis in Southwest Ethiopia.

**Methods:**

A community based cross-sectional study was conducted from February to March 2009 in the Gilgel Gibe field research area. A total of 30,040 individuals 15 years or older in 10,882 households were screened for TB lymphadenitis. Any individual 15 years or older with lumps in the neck, armpits or groin up on interview were considered TB lymphadenitis suspect. The diagnosis of TB lymphadenitis was established when acid fast bacilli (AFB) smear microscopy of fine needle aspiration (FNA) sample, culture or cytology suggested TB. HIV counseling and testing was offered to all TB lymphadenitis suspects. Descriptive and bivariate analysis was done using SPSS version 15.

**Results:**

Complete data were available for 27,597 individuals. A total of 87 TB lymphadenitis suspects were identified. Most of the TB lymphadenitis suspects were females (72.4%). Sixteen cases of TB lymphadenitis were confirmed. The prevalence of TB lymphadenitis was thus 58.0 per 100,000 people (16/27,597) (95% CI 35.7-94.2). Individuals who had a contact history with chronic coughers (OR 5.58, 95% CI 1.23-25.43) were more likely to have TB lymphadenitis. Lymph nodes with caseous FNA were more likely to be positive for TB lymphadenitis (OR 5.46, 95% CI 1.69-17.61).

**Conclusion:**

The prevalence of TB lymphadenitis in Gilgel Gibe is similar with the WHO estimates for Ethiopia. Screening of TB lymphadenitis particularly for family members who have contact with chronic coughers is recommended. Health extension workers could be trained to screen and refer TB lymphadenitis suspects using simple methods.

## Background

TB lymphadenitis is the most common form of extrapulmonary TB [1]. Its epidemiology and diagnostic aspects vary according to the patients’ geographic origin and the burden of TB and HIV infection [2]. Globally the incidence of all forms of TB is decreasing. However, the rate is not similar across all WHO regions. In the Africa region it is decreasing slowly by 1.8% per year [3].

Ethiopia is among the 22 high TB burden countries in the world. The proportion of extrapulmonary TB among newly diagnosed TB patients has been increasing for the last two decades [3]. The WHO and the Ethiopian TB control program estimate that the proportion of extrapulmonary TB among the total number of new TB cases (156,928) is about 32% [4,5]. The majority of extrapulmonary TB cases present with TB lymphadenitis. However, the real burden of pulmonary TB and extrapulmonary TB in Ethiopia is not known at community level. The lack of diagnostic facilities and low quality health services might contribute to low case detection rates [6]. In the absence of a strong surveillance system and well established diagnostic facilities, community-based prevalence studies are good alternatives to estimate the burden of disease. We performed such a study in Southwest Ethiopia to estimate the burden of TB lymphadenitis. To our knowledge, this is the first community-based prevalence survey for TB lymphadenitis in Ethiopia.

## Methods

### Study design, setting and period

**Figure 1:**
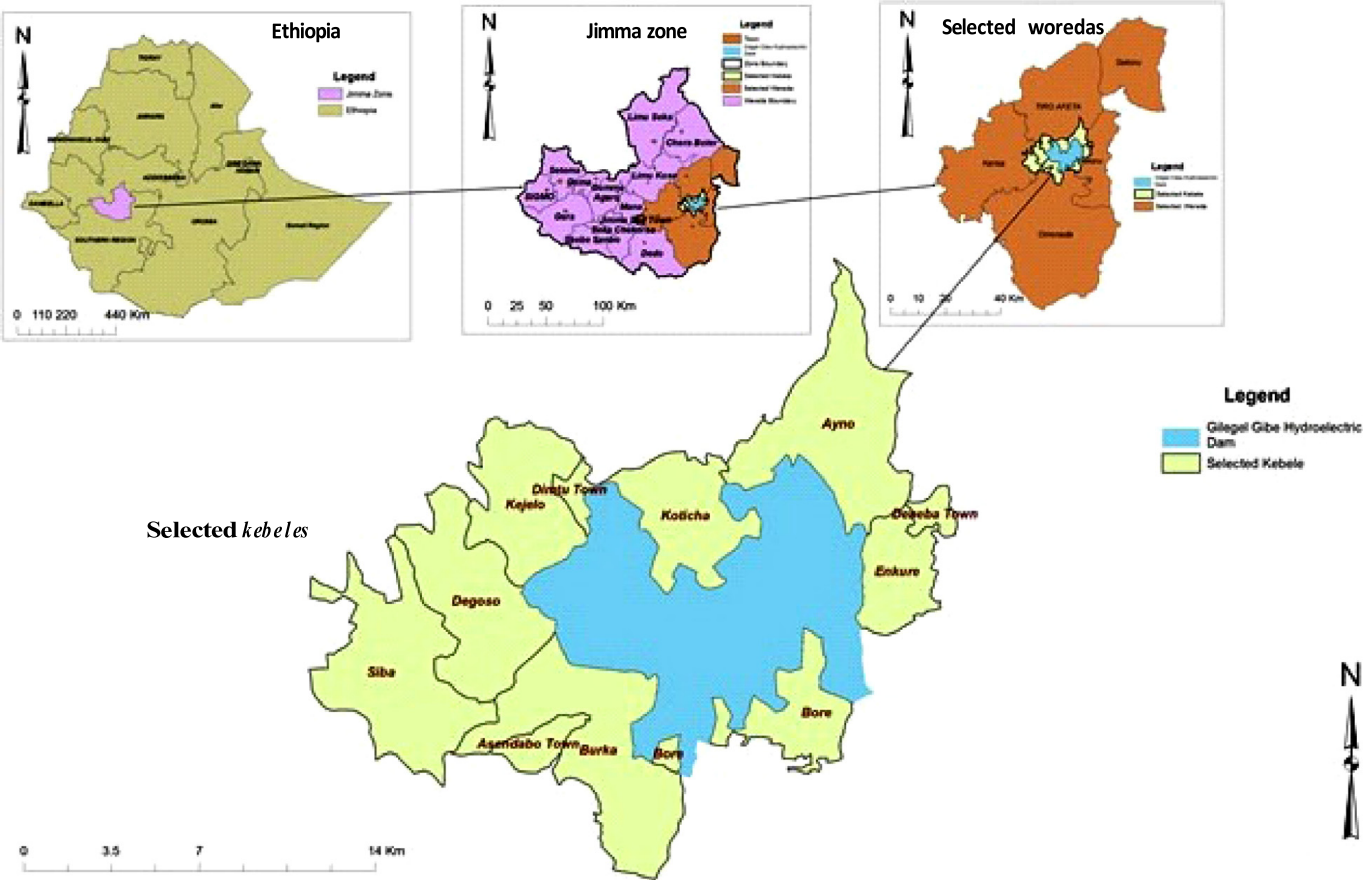
Map of Gilgel Gibe filed research area.

This community-based cross sectional study was conducted from February to March 2009 in Gilgel Gibe Field Research area (Figure 1) which is located in Jimma Zone about 260 kilometers Southwest of Addis Ababa around the reservoir of Gilgel Gibe hydroelectric dam. The site consists of four districts (locally known as *Woredas*): *Sokoru, Omo Nada, Tiro Afata and Qarsa*. Two small towns and eight rural *Kebeles* (smallest administrative units), situated within 10 kilometers of the reservoir of the dam were selected as field research site by Jimma University in 2005. Since then, regular demographic and AIDS mortality surveillances have been undertaken by Jimma University in the research site. At the time of the study, the total population of the field research area was 50,156 individuals in 10,882 households.

### Study population and data collection procedures

At the time of the study the total number of individuals 15 years or older in the study area was 30,040. A questionnaire was prepared in Amharic (local language) to identify TB lymphadenitis suspects in the study area. Trained personnel with high school education visited each household. If the heads of the households were not available during the visit, the data collectors repeatedly visited the same household up to 3 times. Individuals were asked if they had noticed lumps and bumps in the neck, armpits or groin themselves. Only those that respond positively were considered suspects and asked to come the next day to a center to undergo FNA. At the center those TB lymphadenitis suspects were interviewed using a structured questionnaire including questions about his/her socio-demographic situation.

### Sample collection and processing

The FNA was performed by the study pathologists. The diagnosis of TB lymphadenitis was made when smear microscopy, culture and/or cytology suggested TB individually or in combination.

### FNA

FNA was performed using a 22-gauge needle attached to a 10-cc syringe. The aspirates were macroscopically evaluated for caseation. Two smears were prepared from each aspirate and air dried on site. The remainder of the aspirate was washed into 1 ml physiological saline for culture and transported to Jimma University Specialized Hospital in a cold box. FNA samples were kept at −20°C till transported in cold box by postal system to Armauer Hansen Research Institute (AHRI) in Addis Ababa for culture. All samples were transported within a maximum of 5 days.

One slide of a smear was stained with the Ziehl-Neelsen method [7] and examined for the presence of AFB by an experienced laboratory technician. The second smear was stained with Wright stain and cytological analysis was done by the study pathologists at Jimma University. TB lymphadenitis diagnosis by cytology was made by observation of the presence of epitheloid cell granulomas and caseous necrosis with or without Langhan’s giant cells [8,9].

### Culture

At the AHRI, the FNA samples were processed by the standard N-acetyl L-cysteine (NALC)-NaOH method [10] and concentrated at 3000 × *g* for 15 minutes. The sediment was reconstituted to 2.5 ml with phosphate buffer pH 6.8, to make the inoculums for the cultures. Two Lowenstein-Jensen slants, one containing 0.75% glycerol and the other containing 0.6% pyruvate, were inoculated with the sediment and incubated at 37°C. Cultures were considered negative when no colonies were seen after 8 weeks of incubation.

### HIV Screening

All consenting TB lymphadenitis suspects were tested for HIV after counseling. Testing for HIV was done as per Ethiopian national guidelines. HIV screening was performed using the KHB test (Shanghai Kehua Bio-Engineering Ltd, Shanghai, China; 2008). A positive sample was retested using the STAT-PAK test (Chembio Diagnostic System Inc, Medford, NY, USA; 2008).

### Data analysis

Data were double entered using Epi-data version 3.1(Epi-data, Norway, 2006). For analysis, the data were exported to SPSS version 15.0 statistical software (SPSS Inc. Chicago, 2007). Descriptive analysis was done for the socio-demographic variables and prevalence of TB lymphadenitis. Logistic regression was done to assess factors independently associated with TB lymphadenitis in terms of the crude odds ratio and its 95% confidence interval (CI). A p-value less than or equal to 0.05 was considered significant.

### Ethical consideration

The study was approved by the ethical review committees of Jimma University, AHRI and the Institute of Tropical Medicine in Belgium. A workshop was held with the local community leaders, *Kebele* chair persons and Woreda health offices representatives to create awareness about the purpose of the study. Written consent was obtained from the study participants. First the head of the house gave consent. Subsequently depending on the information from the head of the house all the suspects gave consent before administering the questionnaire and undergoing FNA. New TB cases identified were immediately referred to the nearby health facility for treatment.

## Results

### Characteristics of the TB lymphadenitis suspects

A total of 30,040 individuals 15 years or older in 10,882 households were screened for TB lymphadenitis. Complete data were available from 9 *Kelebes* (27,597 individuals 15 years or older) while one *Kebele* was excluded from analysis because of incomplete data. After interview 87 TB lymphadenitis suspects were identified. The mean age (±SD) of the suspects was 33.1 (±11.3) years. Most (72.4%) were women and married (81.6%). Thirty one (35.6%) lived in close contact with animals and 45 (53.5%) used to drink raw milk.

### Prevalence of TB Lymphadenitis

On the basis of FNA smear microscopy for AFB, cytology and culture 16 (18.4%) of the lymph nodes were diagnosed as tuberculous. Smears were positive in 2, culture in 6 and cytology in 13 of the suspects. The microscopic features of FNA cytology of the TB lymphadenitis showed epithelial histocytes with giant cells in 8 cases, epithelial histocytes with caseous necrosis in 2 cases and caseous necrosis alone in 3 cases. Three of these 16 cases were positive by both culture and FNA cytology. The mycobacteria isolated in 6 cases were identified as *Mycobacterium tuberculosis* based on RD typing*.* The remaining 10 cases that were negative on culture but positive on smear and/or cytomorphology were also considered to be tuberculous. The distribution of TB lymphadenitis cases per *kebele* is indicated in Table 1. The prevalence of TB lymphadenitis was 58.0 per 100,000 people (16/27,597) (95% CI 35.7-94.2). Other diagnosis of the lymph nodes include: reactive lymphadenitis (57), pyogenic lymphadenitis (6), benign mesenchymal neoplasia (6) and non-specific lymphadenitis (5).

**Table 1:** **Distribution of TB lymphadenitis cases in the study*Kebeles*of Gilgel Gibe Field Research Center, Southwest Ethiopia**

**Kebele**	**Total population surveyed**	**TB lymphadenitis**	**95% CI**
		**n (prevalence per 100,000 people)**	
Siba	2893	1 (34.6)	6.1-195.5
Asandabo	4493	0 (0)	0-85.4
Burka	3539	1 (28.3)	5.0-159.9
Kajelo	3409	2 (58.7)	16.1-213.7
Koticha	2342	1 (42.7)	7.5-241.5
Ayino	3382	2 (59.1)	16.2-215.4
Enkure	2048	3 (146.5)	49.8-429.8
Bore	2065	0 (0)	0-185.7
Danaba	3426	6 (175.1)	80.3-381.6

*CI* Confidence interval.

### Epidemiological factors associated with TB lymphadenitis

TB lymphadenitis was more frequent in females (86.5 per 100,000) and age group 35–44 (90.4 per 100,000 people) followed by 45 years or older (71.9 per 100,000 people). The differences in the prevalence of TB lymphadenitis with respect to sex and age group were not statistically significant (Table 2). Other personal characteristics associated with TB lymphadenitis are described in Table 3. Only contact with a person with chronic cough was significantly associated with TB lymphadenitis (OR 5.58, 95% CI 1.23-25.43).

**Table 2:** Prevalence of lymphadenopathy and tuberculous lymphadenitis per 100,000 people by sex and age in Gilegl Gibe, south west Ethiopia

**Variable**	**Total population**	**Lymphadenopathy n(prevalence)**	**TB Lymphadenitis n (prevalence)**	**OR [95% CI]**
**Sex**				
Male	13718	24 (174.9)	4 (29.2)	1
Female	13879	63 (453.9)	12 (86.5)	2.97 [0.97- 9.20]
**Age**				
15-24	10482	16 (152.6)	3 (28.6)	1
25-34	7129	30 (420.8)	5 (70.1)	3.43 [0.89- 13.28]
35-44	4423	28 (633.1)	4 (90.4)	2.37 [0.48- 11.75]
> = 45	5563	13 (233.7)	4 (71.9)	1.89 [0.38- 9.34]

*OR* Odds ratio, *CI* Confidence interval.

**Table 3:** Characteristics of persons with tuberculous and non-tuberculous lymphadenitis, Gilgel Gibe, southwest Ethiopia

**Characteristics of persons**	**TB status**		**OR [95% CI]**
	Positive n (%)	Negative n (%)	
**Sex**			
Male	4 (16.7)	20 (83.3)	1.18 [0.34-4.1]
Female	12 (19.0)	51 (81.0)	1
**Age**			
15-24	3 (18.8)	13 (81.2)	1
25-34	5 (16.7)	25 (83.3)	0.87 [0.18-4.21]
35-44	4 (14.3)	24 (85.7)	0.72 [0.14-3.73]
> = 45	4 (30.8)	9 (69.2)	1.93 [0.34-10.77]
**Occupation**			
Farmer	11 (19.6)	45 (80.4)	0.69 [0.40- 4.06]
Non-farmer	5 (16.1)	26 (83.9)	1
**Monthly income**			
<=400 birr (<=34 USD)	10 (21.3)	37 (78.7)	1.35 [0.14- 12.92]
>400 birr (>34 USD)	1 (16.7)	5 (83.3)	1
**Contact with a chronic cougher**			
Yes	4 (50.0)	4 (50.0)	5.58 [1.23- 25.43]*
No	12 (15.2)	67 (84.8)	1
**Contact with cattle**			
Yes	6 (19.4)	25 (80.6)	1.10 [0.36- 3.39]
No	10 (17.9)	46 (82.10)	1
**Drink uncooked milk**			
Yes	10 (21.3)	37 (78.7)	1.53 [0.50- 4.67]
No	6 (15.0)	34 (85.0)	1

*OR* Odds ratio, *CI* Confidence interval, *USD* United Sates Dollar, *p < 0.05.

### Characteristics of the lymph nodes associated with TB lymphadenitis

Lypmh node characteristics affecting positivity for tuberculous lymphadenitis are described in Table 4. Among the lymph nodes characteristics only caseous FNA samples were more likely to be from persons with TB lymphadenitis (OR 5.46, 95% CI 1.69-17.61).

**Table 4:** Characteristics of the lymph nodes in persons with tuberculous and non-tuberculous lymphadenitis, Gilgel Gibe, southwest Ethiopia

**Characteristic of the lymph node**	**TB status**		**OR [95% CI]**
	Positive n (%)	Negative n (%)	
**Duration**			
<=6 weeks	8 (25.0)	24 (75.0)	2.28 [0.71- 7.36]
7-12 weeks	2 (25.0)	6 (75.0)	2.28 [0.37- 14.0]
>12 weeks	6 (12.8)	41 (87.2)	1
**Tenderness**			
Tender	2 (18.2)	9 (81.8)	0.98 [0.19- 5.07]
Non-tender	14 (18.4)	62 (81.6)	1
**Number of lymph nodes**			
Single	5 (12.2)	36 (87.80)	1
Few	2 (20.0)	8 (80.0)	1.80 [0.30- 11.0]
Multiple	9 (25.0)	27 (75.0)	2.40 [0.72- 8.0]
**Size**			
1-4 cm	11 (15.5)	60 (84.5)	1
5-10 cm	5 (31.2)	11 (68.8)	2.48 [0.30- 11.0]
**Mobility**			
Mobile	15 (21.4)	55 (78.6)	1
Non-mobile	1 (5.9)	16 (94.1)	0.23 [0.03- 1.87]
**Condition**			
Soft	4 (17.4)	19 (82.6)	
Firm	10 (16.9)	49 (83.1)	
Matted	2 (66.7)	1 (33.3)	
Draining sinus	0 (0.0)	1 (100.0)	
Fluctuant	0 (0.0)	1 (100.0)	
	**Lymph node involved**		
Cervical	12 (18.5)	53 (81.5)	1
Axiliary	3 (27.3)	8 (72.7)	1.66 [0.38- 7.19]
Inguinal	1 (11.1)	8 (88.9)	0.55 [0.06- 4.84]
Others	0 (0.0)	2 (100.0)	
**Nature of FNA specimen**			
Caseous	8 (42.1)	11 (57.9)	1
Non-caseous	8 (11.8)	60 (88.2)	5.46 [1.69- 7.61]*

*OR* Odds ratio, *CI* Confidence interval, *p < 0.05.

## Discussion

Extrapulmonary TB is a significant health problem worldwide because of difficulties in its diagnosis and in monitoring its treatment. The proportion of extrapulmonary TB among all TB cases varies from country to country. Of the 22 high burden countries the highest proportion was reported from Cambodia (34.2%) and the lowest from China (0.69%) [5]. The extrapulmonary manifestation of TB is prevalent in 10-34% of non-HIV cases while it occurs in 50-70% of patients co-infected with HIV [11]. Ethiopia reports the third highest number of extrapulmonary TB globally (50,417) [5]. In the current study, we assessed TB lymphadenitis, the major form of extrapulmonary TB, in a rural community in Ethiopia.

In this study the diagnosis of TB lymphadenitis was established when FNA smear microscopy for AFB, culture and/or cytology reported positive. In fact cytology suffers from low number of cells that are all in dispersion rather than organized tissue as in biopsies. Nevertheless, cytology is specific when compared against combined criteria [3].

The prevalence of pulmonary TB was 76.1 per 100,000 people in the same study population during the same study period [12]. It is higher than the prevalence of TB lymphadenitis as reported here. TB lymphadenitis accounted for 43.2% (95%CI: 28.7-59.1) of all forms of TB diagnosed in the study setting. A previous report from Ethiopia also indicated that TB lymphadenitis accounted for 40% of total TB cases in a rural health center [13]. WHO estimates for Ethiopia that extrapulmonary TB accounts for roughly one third of the new cases of TB in the country [5]. However, it is not possible to compare the findings from our study with the national figure of the WHO estimates because of two main reasons. First, the WHO data were derived from health institution based reports. Second, our study did not investigate all forms of extrapulmonary TB. Investigating the different parameters to identify risk factors for TB lymphadenitis in areas of high and low proportions in the country is recommended.

It has been reported that TB case detection remains very low in Ethiopia [14]. Indeed, in our study all the TB lymphadenitis cases were newly diagnosed. The efforts to improve TB case detection in the country should be strengthened. It is logical that the health care seeking behavior could be low for TB lymphadenitis compared with that of pulmonary TB. This is mainly because pulmonary TB has a higher case fatality rate than TB lymphadenitis and the latter is mainly of cosmetic concern at least initially, which takes lower priority in rural communities with poor access to health facilities. Nevertheless, the health extension workers should be trained to identify not only pulmonary but also extrapulmonary TB suspects in the community and link them to health facilities.

A contact history with a chronic cougher was highly related with the odds of having TB lymphadenitis. This partly indicates that there is transmission going on in the community although further study may be needed to unravel the transmission dynamics. Moreover, this might indicate that the route of acquisition of lymph node TB is probably the same as pulmonary and can partly explain why *Mycobacterium bovis* was not detected. Other studies have reported a predominance of *Mycobacterium tuberculosis* in TB lymphadenitis in Ethiopia [15,16] although bovine transmission would be expected where raw milk is consumed.

Cervical lymph nodes were the prominent sites involved in our study supporting previous reports [17-19]. Studies have reported that women were more likely to be positive for TB lymphadenitis compared with men [20-25]. Moreover, it has been suggested that in male dominated communities, where women experience poorer living conditions, young females generally notice differences in their appearance earlier than males [26]. In our study, however, the rate of TB lymphadenitis was not significantly different between the two sexes. Our previous finding from the study area on health seeking behavior also did not find a difference in gender [27]. The age group 35–44 years and FNA samples from axillary lymph nodes has shown the largest proportion of cases but difference in prevalence in terms of age group and location of the lymphnodes was not statistically significant.

Previous studies have suggested that the extrapulmonary forms of TB could be attributed to HIV co infection [18,28]. In Ethiopia the HIV prevalence for rural areas was estimated to be 0.9% [29]. However, in our case all the TB suspects were HIV negative. This suggests that apart from HIV either mycobacterial strains or host factors may play an important role in TB lymphadenitis in this community. Recent reports elsewhere identified that single nucleotide polymorphisms rs4893980 on gene PDE11A of chromosome number 2, rs10488286 on gene KCND2 of chromosome number 7 and rs2026414 on gene PCDH15 of chromosome number 10 in humans were associated with extrapulmonary tuberculosis [30]. In this study we did not do the typing for host genetic factors predisposing for TB lymphadenitis.

The strength of our study was that it has tried to address the neglected component of TB at community level. However, our study was not without pitfalls. We probably underestimated the prevalence of tuberculous lymphadenopathies because individuals were not systematically examined for lymphadenopathies and culturing of the samples were delayed due to transport problems. Moreover, the methods that we used for the laboratory diagnosis of lymph node TB are not the most sensitive and specific. We did not use PCR which may have high sensitivity in detection of lymph node TB [31]. These all limitations in combination may have resulted in under estimation of the lymph node TB prevalence in communities living in Gilgel Gibe research site.

## Conclusions

The prevalence of TB lymphadenitis was 58 per 100,000 people in Gilgel Gibe. Screening of TB lymphadenitis particularly for family members who have contact with chronic coughers is recommended. Health extension workers could be trained to screen and refer TB lymphadenitis suspects using simple methods.

## Competing interest

The authors declared that there is no competing interest.

## Authors’ contribution

GA was involved in the conception and design of the study, coordinated the field work, analyzed the data and drafted the manuscript. AD was involved in the conception, design of the study, field work and review of the article. LA was involved in the design and reviewed the article. AA, FD, KW, CJ, MT, JS, AAseffa and MB participated in the design, field work and reviewed the article. MB and AB participated in lab work and reviewed the article. RC participated in the design**,** critically reviewed and approved the article. All authors read and approved the final manuscript.

## Pre-publication history

The pre-publication history for this paper can be accessed here:

http://www.biomedcentral.com/1471-2458/12/504/prepub
